# Systematic review of the effects of agricultural interventions on food security in northern Ghana

**DOI:** 10.1371/journal.pone.0203605

**Published:** 2018-09-07

**Authors:** Michael Osei Adu, David Oscar Yawson, Frederick Ato Armah, Ernest Ekow Abano, Reginald Quansah

**Affiliations:** 1 Department of Crop Science, School of Agriculture, College of Agriculture and Natural Sciences, University of Cape Coast, Ghana; 2 Department of Environmental Science, School of Biological Science, College of Agriculture and Natural Sciences, University of Cape Coast, Ghana; 3 Department of Agricultural Engineering, School of Agriculture, College of Agriculture and Natural Sciences, University of Cape Coast, Ghana; 4 Department of Biological, Environmental & Occupational Health Sciences, School of Public Health, College of Health Sciences, University of Ghana, Accra, Ghana; TNO, NETHERLANDS

## Abstract

**Background:**

Food insecurity and poverty rates in Ghana are highest in the districts from latitude 8° N upwards. These have motivated several interventions aimed at addressing the food insecurity via promoting agricultural growth. An assessment of the overall impact of these interventions on food security is necessary to guide policy design and future interventions.

**Methods and findings:**

A systematic review was used to assess the cumulative evidence of the effect of development interventions, implemented from 2006 to 2016 on food security, especially in Northern Ghana. Information were retrieved from over 20 Government and non-Governmental organisations through online search and actual visits. The number of studies included in systematic review was 22. The study showed that a large number of interventions have been implemented in Northern Ghana over the study period. Access to quality extension services, training and capacity building was a major intervention strategy. About 82% of studies considered increasing production but only 14% of the studies reported on changes in yield. About 42% of the included studies used market access as a strategy but about 44% reported increase in incomes of beneficiaries (with only seven studies providing numerical evidence for this claim). The ranking of frequency of intervention strategies was in the order *extension and capacity building > production > postharvest value addition > water and irrigation facilities > storage facilities > input supply*. A substantial number of the studies had no counterfactuals, weakening confidence in attributing impacts on food security for even the beneficiaries.

**Conclusions:**

It is concluded that evidence for impacts of the interventions on food security was weak, or largely assumed. A logical recommendation is the need for development partners to synchronise their measurement and indicators of food security outcomes. It is also recommended that some food security indicators are explicitly incorporated into intervention design while bearing in mind the potential need for counterfactuals.

## Introduction

The food and agriculture sector provides great opportunities for poverty reduction and development in low income countries. The importance of food security was recognized in both the Millennium Development Goals (MDG 1) and the post-MDG Sustainable Development Goals (SDG 2). Poverty and food insecurity are strongly linked [1] and interact in complex ways to deliver undesirable development outcomes especially in low income countries. This strong relationship arises from the need to access food physically and economically, and the large dependence of livelihoods, incomes and economic growth on agriculture. As a result, the agricultural sector is an attractive locus for reducing poverty and hunger in low income countries. A review by [2] indicated that low income is a major cause of food insecurity. At household level, access to food can occur via direct production or purchases from food markets. Hence, increasing the productive capacities of households and communities that depend directly on agriculture for food and/or livelihoods is perceived to be an effective route to reducing poverty and food insecurity simultaneously [3].

Moreover, even though the overall contribution of agriculture to Gross Domestic Product (GDP) is decreasing in several low income countries, agriculture remains the largest employer in most African countries [4]. In such countries, smallholder farmers account for the largest share of agricultural production. Often, these smallholder farmers have poor resource base, poor access to markets and are vulnerable to poverty and food insecurity. Consequently, agricultural development interventions are fundamental to reducing poverty and food insecurity at varying spatial and temporal scales across Africa. Agricultural development interventions can also be used as instruments for building long term competitiveness and resilience in local to national food systems in the face of rapidly changing socio-ecological conditions [5].

Globally, several agricultural development interventions, with varying goals and approaches, have been carried out by national governments, development partners, and non-governmental organizations (NGOs) as part of overall national or regional development strategies, with mixed results and outcomes. Because food security has become a key theme in international development cooperation [6], agricultural development interventions abound in Africa, ranging from input supplies, land tenure, soil and water management, agronomic management, market access, postharvest handling, value addition, skills training and knowledge transfers, and financial assistance. However, there can be a gap between successful project implementation (based on the intervention logic and measures of success) and practical, verifiable impacts of agricultural development interventions. A broad systematic review by [6] showed that quantitative evidence of actual, direct effects of interventions on food security is limited. Given the importance of agricultural development interventions for overall poverty reduction, food security and development, national and/or subnational assessment of the impacts of interventions on food security is crucial to inform subsequent designs and food security policy.

Ghana is a West African country with 10 administrative regions. Agriculture employs over 50% of the labour force and about 90% of farm holdings are less than two hectares in size [7]. These smallholder farmers account for the bulk of food production under rain-fed conditions. The Government of Ghana defines food security as “good quality nutritious food, hygienically packaged and attractively presented, available in sufficient quantities all year round and located at the appropriate places at affordable prices” [7]. Improvement in food security and emergency preparedness are core components of the agricultural development and poverty reduction strategy of the Government of Ghana as captured in the Food and Agriculture Sector Development Policy (FASDEP II) [8]. Ghana is rich in natural resources and has made substantial progress in reducing poverty and food insecurity in the past decade. However, the twin problem of poverty and food insecurity remains a major development challenge, particularly in the three northern regions (Upper East, Upper West and Northern, collectively referred to as Northern Ghana in this paper). The Northern Ghana is known to have the highest prevalence of poverty and food insecurity in Ghana [9]. For example, the Northern Ghana has the highest rates of stunting and wasting [10]. Livelihoods in Northern Ghana depend largely on agriculture or natural resources [11]. Even though there is huge potential for food production in this zone, the ecological conditions (savannah/sahelian conditions) combine with poor resource base to reinforce low agricultural productivity, food insecurity and poverty. Consequently, there has been a disproportionately higher concentration of agriculture-related international development interventions in Northern Ghana compared to all other regions. Information on the pooled effect of the numerous interventions on food security in this zone in particular, and in Ghana generally, is lacking but crucial for identifying gaps and overlaps, and to inform future interventions and policy actions.

Substantial body of information has been generated through the agriculture-related development interventions. While this body of information could guide current and future food security efforts, it is fragmented, scattered and/or difficult to access. The current study therefore systematically reviewed agricultural interventions in Northern Ghana to generate evidence of impacts on food security mainly at the household level. The study was aimed at synthesizing the fragments of information on food security interventions to generate a pooled evidence and increase its access and use for policy actions and future intervention designs. Specifically, this paper sought to systematically review the pooled effect of agriculture-related development interventions in Northern Ghana on food security from 2006 to 2016, and to synthesize the available information into a format usable for evidence-based policy and intervention design. We chose 2006–20016 for this review because within this period, food prices nearly doubled and the number of people suffering from hunger and undernourishment soared, leading to a renewed interest in food security as one of the key themes in international development co-operation [6]. Moreover, we sought to capture the latest trends in agricultural development interventions on food insecurity and to cover more recent than former years. We also considered the 10-year time restriction employed here as acceptable to enable the cumulative impact of interventions to be measured.

## Methodology

The present study adapted the protocol developed by the Campbell Collaboration, which was used for a similar study by the Policy and Operations Evaluation Department of the Ministry of Foreign Affairs of the Netherlands [6]. Key components of the approach adopted here included: (i) an explicit search strategy involving a desktop search to retrieve literature from scientific databases; (ii) visits to identifiable organizations to collect available literature (such as reports) that is either unpublished or difficult to access; (ii) clear inclusion/ exclusion criteria; and (iii) systematic coding and analysis of included studies.

### Data sources

Two types of data sources were utilised: (i) a desktop search involving searching for information online and (ii) visits to organisations to retrieve information. To begin with, a shortlist of organizations considered as hugely involved in food security interventions was created. This list was informed to a large extent by the Ghana agriculture sector development partners’ coverage map produced by the Monitoring, Evaluation and Technical Support Services in Ghana (METSS- http://www.metss-ghana.k-state.edu/maps.html). The list guided desktop search and visits for documents that are unpublished or difficult to access. The visits to the organizations also provided opportunity for snowball sampling of other organizations and information retrieval. For the desktop search, peer-reviewed scientific publications were searched using key words, probable titles, and logical operators and filtering techniques. Scientific databases including Google Scholar, Web of Science, Science Direct, RefSeek, and Scopus were searched. The string for searching key words in title and topic was: "Ghana AND food security" AND "impact" AND ("agricultur*" OR "production" OR "production value" OR "production costs" OR "markets" OR "trade" OR "prices" OR "safety net" OR "women" OR "gender" OR "environment" OR "finance" OR "value chain" OR “Intervention” OR “Livelihood”). To reduce positive-result publication bias, and provide more complete information, the so-called ‘grey literature’ was also searched to identify evaluation reports available from the websites of identified organizations and institutions, including the portals of donor governments. The grey literature search included web portals and general internet search using different search engines and strategies. These included the Africa South of the Sahara Food Security Portal and Ebrary knowledge repository both hosted by the International Food Policy Research Institute (IFPRI), the essential electronic agriculture library (TEEAL), access to global online research in agriculture (AGORA), and online access to research in environment (OARE) led by the United Nations Environment Programme (UNEP). The second part of information retrieval involved actual visits to various organizations and institutions to access unpublished literature such as annual reports, evaluation reports, dissertations and thesis on food security in Northern Ghana.

### Inclusion/exclusion criteria and evaluation criteria

Generally, criteria related to the evaluation of the ‘quality’ and the ‘content’ of retrieved documents were developed a priori. A document was included in the study if it fulfilled the following a priori eligibility criteria: (i) is an original study; (ii) reports on any one or more of the following outcomes: food security, food insecurity, food stability, food availability, food utilization and food accessibility, food security intervention, impact evaluation of food security intervention; and iii) presents data on household level food security. Our exclusion criteria were a) a study on food security not of relevance to Ghana or Northern Ghana, b) a study on food security outside the time period of this study (2006–2016), and c) literature that did not directly address the objectives of this study.

### Quality of the document

**Fig 1:**
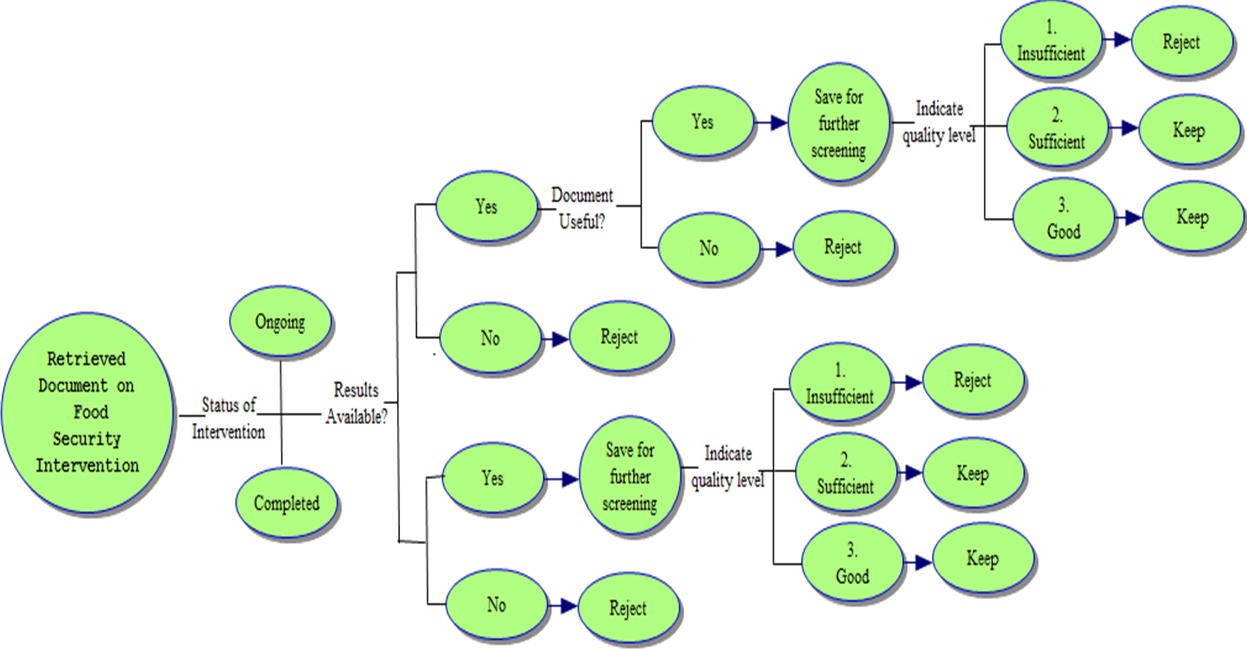
Flow chart for the criteria related to the quality of the document.

Quality of retrieved documents was assessed using the process summarized in Fig 1. Based on quality, documents were categorized as (i) Good; (ii) Sufficient; or (iii) Insufficient. A document was classified as ‘Good’ if it was a full report and results were attributed to the intervention by a plausible counterfactual analysis (e.g.: clear description of intervention strategy; there is both a comparison of ‘before-after’ intervention or a comparison of ‘with-without’ intervention; there is clarity of the problem definition and research questions, etc.). A document was classified as ‘Sufficient’ if it was a full document, a project profile or summary with some details of the intervention, preferably with objectives and some results but without counterfactual analysis. An ‘Insufficient’ document had vague description of intervention, no results, no comparison to counterfactuals, and no reliability of the information source. Documents classified as ‘Good’ or ‘Sufficient’ were kept for further screening based on content of the document, whereas documents classified as ‘Insufficient’ were rejected at this stage.

### Content of document

**Fig 2:**
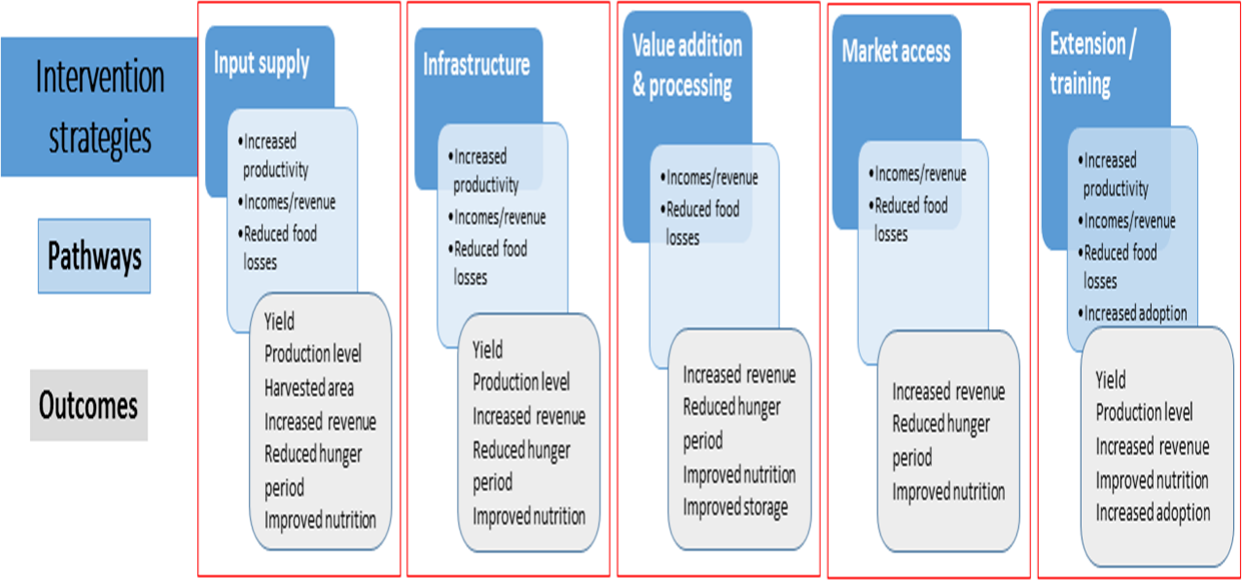
Intervention strategies, pathways and outcomes of food security used in this study.

Given that food security is a very broad term encompassing many disciplines, the scope of this study was limited to the analysis of five intervention strategies and their pathways to food security. Emphases were also placed on studies that had disaggregated data based on gendered household headship. The five intervention strategies prioritized were related to: (i) *inputs supply*: interventions that support agricultural production directly, through provision of seeds, fertilizers, land, other agrochemicals etc.; (ii) *infrastructure*: including water, irrigation, storage facilities etc.); (iii) *value addition / processing*; (iv) *market access*: interventions operationalized in both the input and output markets, interventions that provided or enhanced markets to farmers, or interventions that sought to make markets more efficient, to open up markets for consumers, or to protect domestic production; and (v) *extension and training*: interventions targeting the training of extension officers and/or farmers or delivering improved or targeted extension services and access to finance. Obviously, these food security intervention strategies would have more than one outcome. The simplified constructed pathways and their respective outcomes considered in this study are presented in Fig 2. Some outcomes were specific for some intervention strategies but others embodied multiple strategies.

### Coding of reports, analyses of included studies

The retrieved studies were coded to capture the intervention strategies, outcomes and characteristics. A coding sheet was used to extract the essential information from each selected document for further analysis. A document could also be rejected at this stage if upon careful scrutiny it lacked the relevant intervention strategies and outcomes. Descriptive statistics and non-parametric tests were used to analyse the included studies. An investigator performed the online literature search but all five authors (raters) were involved in the assessment of manuscripts for inclusion/exclusion, data extraction and in the coding of included studies. The raters preliminarily coded a number of trial documents and met a predefined competency criterion before proceeding to the extraction and coding of the actual studies. In addition to the primary extraction and coding, an independent coder rated an adequate representation of the included studies to allow for the generation of inter-rater agreement data and provide an estimate of the reliability of the extraction and coding process. The a priori threshold of inter-rater agreement in this study was set to 80%. That means that, four out of the five authors must agree on the rating of each of the studies reviewed and on the data extracted from it. In all cases except two, the percentage of agreement was acceptable. The ensuing disagreements on the two papers were resolved by building consensus whereby the coders discussed these discrepancies and consequently resolved them. Non-parametric tests (Pearson chi-square statistic) were used to determine whether the observed differences in the regions of the study, duration of intervention, production level, yield, number of extension services rendered and specific food security indicators for any pair of measure were statistically significant. In instances, where they were significant, the effect size was estimated using Cramer’s V. Summaries of some of the included studies, their claims or results were also discussed. It must be pointed out that this study also intended to conduct meta-analysis to quantify impacts of the interventions if the retrieved documents had the appropriate data.

## Results

### Description of included studies

**Fig 3:**
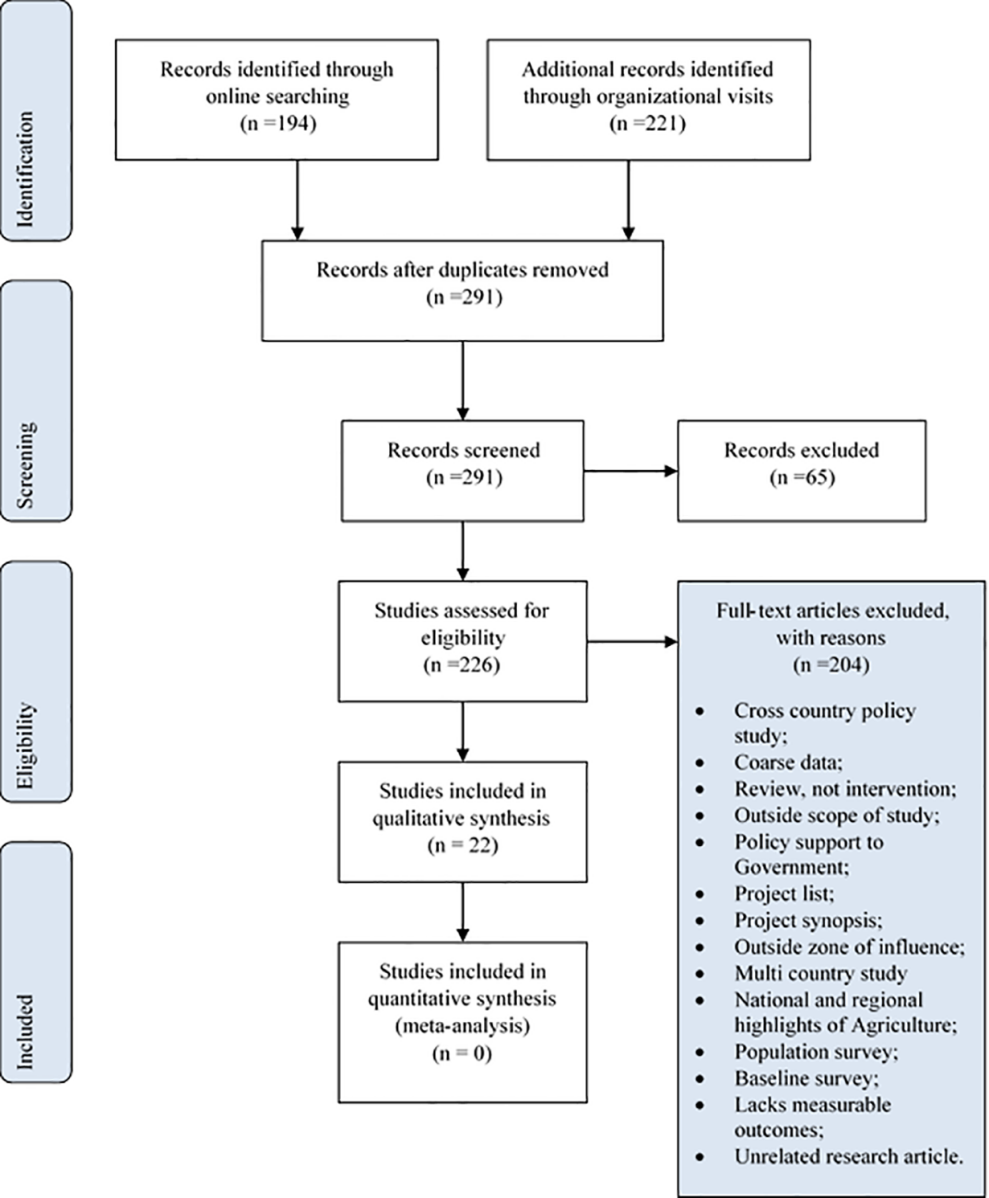
PRISMA flow diagram of review of studies on food security interventions in Ghana.

Information were retrieved from over 20 Government and non-Governmental organisations through online search and actual visits (Table 1). The number of records retrieved from online and those retrieved from visit to organisations were 194 and 221, respectively (Table 1 and Fig 3). Fig 3 also shows PRISMA flow diagram (S1 File) of the review detailing the number of studies during the subsequent screening and selection procedures. Full-text articles assessed for eligibility and those excluded, with reasons were 226 and 204, respectively (Fig 3). The number of studies included in qualitative synthesis is 22. No study was included in quantitative synthesis (meta-analysis) as originally intended because the studies evaluated either did not have the relevant data for meta-analysis or had many different quantitative and qualitative indicators, which were difficult to normalize (Fig 3).

**Table 1:** Number of studies found after the visits and initial key word search and after the first screening on title and abstract.

Source of Record*	Records Retrieved from Online	Records Retrieved from Visit to Organisation
ACDEP	-	2
ACDI VOCA	2	-
AfDB	2	-
AGRA	12	5
CIDA	54	4
Concern Universal	-	3
Conservation Alliance	-	7
CSIR-SARI	-	10
IFPRI	8	-
IPA	15	1
JICA	-	3
MOFA	-	5
NRGP	-	49
NSAICU	-	28
Other Organisations**	39	-
University of Ghana	-	54
USAID / METSS	57	40
USAID RING	-	6
WFP	-	4
World Bank	5	-
**Total**	**194**	**221**

*Abbreviations: ACDEP: Association of Church-Based Development; ACDI/VOCA: Agricultural Cooperative Development International and Volunteers in Overseas Cooperative Assistance; AfDB: African Development Bank; AGRA: Alliance for Green Revolution in Africa; CIDA: Canadian International Development Agency; CSIR-SARI: Council for Scientific and Industrial Research-Savannah Agricultural Research Institute; IFPRI: International Food Policy Research Institute; IPA: Innovations for Poverty Action; JICA: Japan International Cooperation Agency; MoFA: Ministry of Food and Agriculture; NRGP: Northern Rural Growth Program; NSAICU: Northern Sector Agriculture Investment Coordination Unit; USAID / METSS: United States Agency for International Development / Monitoring Evaluation and Technical Support Services; USAID / RING: United States Agency for International Development / Resiliency in Northern Ghana; WFP: World Food Programme.

**The names of the "other organisations" from which reports/literature were obtained are CARE Ghana, Prolinnova-Ghana platform, Innovations for Poverty Action (IPA), International Fund for Agricultural Development (IFAD), Mennonite Economic Development Associates (MEDA), Millennium Challenge Account (MCA) Ghana programme, Canadian Hunger Foundation, Oxfam America, World vision international and Australian Aid.

Of all the interventions reported in the included studies (S1 Table), 82% reported having supplied inputs (which comprised seeds, fertilizers, land, agro-chemicals) as a strategy (Table 2). However, for all studies that used input supply as intervention strategy, only 4% actually supplied seeds while 11% actually supplied two or more inputs (such as seeds and fertilizers). About 42% of the included studies focused on market access (Table 2). Extension and capacity building featured in all the studies included. Approximately 30% reported postharvest value addition. Further, some studies undertook interventions related to infrastructure. Of these, 19% of the studies focused on water and irrigation facilities while 11% were on storage facilities (Table 2).

**Table 2:** Summary statistics on intervention strategies and outcomes.

Studies	n = 27*	
Intervention strategy reported	Yes (%)	No (%)
*Input Supply*	81.5	18.5
Seed systems	4.1	95.9
Two or more inputs	11.2	88.8
*Market access*	42.3	57.7
*Extension training/capacity building*	100.0	0.0
*Post-harvest value addition*	29.6	70.4
*Infrastructure*		
Water and Irrigation	19.2	18.8
Storage facilities	11.4	88.6
***Intervention outcome***		
Changes in yield	13.9	87.1
Production level	41.3	58.7
Increase in revenue	44.2	45.8
Increase in technology adoption	73.9	26.1
Improved nutrition	0.0	0.0
Reduced hunger period	7.0	83.0

*Sample size (n) = 27 because some studies had provided information on multiple crops and these crops were coded independently for our analysis. Original number of included studies was 22.

Main strategies are italicised.

With regard to outcomes measured, 14% of the studies measured change in yield. All of these studies reported increase in yield (Table 2). Similarly, 41% of the studies reported increase in the level of production at farm or communal scale while 44% reported increase in revenues of intervention participants. Only 7% of the interventions measured increase in duration of food availability at household level (i.e. reduced hunger period) whereas 74% reported increase in adoption of technology from the extension, training and capacity building interventions. None of the studies measured meals eaten per day by intervention group or improved nutrition. The studies scarcely decomposed outcome measurement based on household head.

The highest number of strategies simultaneously considered by a single food security intervention project or study was four (Table 3). About 17% of studies at the national level focused on only one intervention strategy, whereas 67% and 16% of studies at the national level focused simultaneously on two and three intervention strategies, respectively. None of the national level studies focused on four intervention strategies simultaneously in a single study. Most of the interventions in the three northern regions focused simultaneously on three strategies (Table 3). Studies carried out jointly in the Brong Ahafo and Volta regions (included because of northern districts of Brong Ahafo region) focused on four intervention strategies simultaneously in a single project. Generally, interventions in northern Ghana focused on two or three strategies.

**Table 3:** Number of intervention strategies considered by various food security intervention projects across Ghana based on studies evaluated.

Region of intervention	Number of simultaneous intervention strategies targeted (%)				
	One	Two	Three	Four	
National	17	67	16	0	*Pearson chi* ^*2*^*(15)*
Northern	0	100	0	0	*= 33*.*2321;*
Brong Ahafo & Volta	0	0	0	100	*Pr = 0*.*004;*
Northern & Upper East	0	0	100	0	*Cramér's V =*
Northern, Upper East & Upper West	14	0	86	0	*0*.*7096*
Northern & Brong Ahafo	0	50	50	0	

Multiple interventions correspond to two or more of the intervention strategies outlined earlier or indicated in Fig 2 had been applied simultaneously in a single project.

The chi-squared statistic reported for the number of food security intervention strategies implemented both regionally and nationally based on the studies examined firmly rejects the hypothesis that region and number of food security intervention strategies are independent (Table 3). The Cramer’s V statistic obtained for inter-correlation between region where the intervention was implemented and the number of intervention strategies undertaken was 0.71 (Table 3).

The analysis also suggested that there is some association between how long an intervention lasted (i.e. duration of the food security intervention) and the level of success of capacity building through provision of extension services (i.e. results obtained following an extension training). The chi-squared statistic reported rejects the hypothesis that extension training is independent of the duration of the intervention. On the whole, adoption of technology introduced by extension services was higher for interventions that lasted longer. The effect size was also large based on the Cramer’s V value of 0.67 (Table 4). Higher production levels were associated with interventions that lasted longer compared with those with shorter duration (Table 5). The association between the two variables was also very strong. There were however no significant relationships between duration of food security intervention and yield (*Pearson chi*^*2*^ *(2) = 1*.*3114; Pr = 0*.*519*), income of households (*Pearson chi*^*2*^*(2) = 1*.*0867; Pr = 0*.*581*) and duration of the year in which food was available in the household *(Pearson chi*^*2*^*(2) = 3*.*5133; Pr = 0*.*173*). Interventions that targeted productivity of agricultural households were strongly associated with higher yields (*Pearson chi*^*2*^ *(1) = 13*.*1250; Pr = 0*.*000*).

**Table 4:** Association of number of extension training and duration of food security intervention.

Outcome of extension training (%)	1–3 years	3–5 years	>5 years	*Pearson* *Chi* ^*2*^*(2)*	*Pr*.	*Cramér's V*
Increase in adoption	10	79	11	9.3947	0.009	0.6689
None observed	100	0	0			

**Table 5:** Association of production level and duration of food security intervention.

Production level (%)	1–3 years	3–5 Years	>5 years	*Pearson* *Chi* ^*2*^*(2)*	*Pr*.	*Cramér's V*
Increase	12	88	0	7.1604	0.028	0.7152
None observed	67	17	16			

### Impacts on yield and income

**Fig 4:**
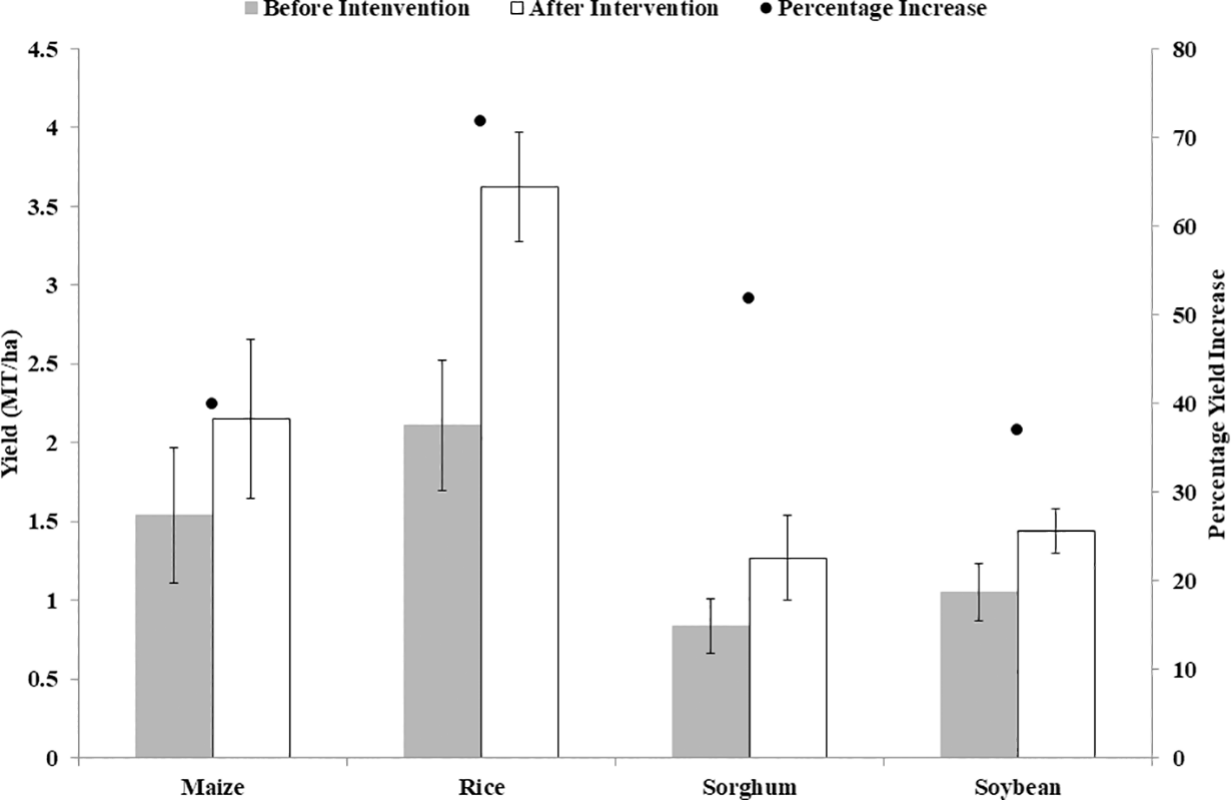
Mean crop yields before and after interventions.

Basic analysis of yield data in the studies that reported yields before and after interventions is shown in Table 6 for four main crops (maize, rice, sorghum and soybean). For studies that reported changes in yields before and after interventions, large increases in yields were reported (Fig 4). Rice had the largest percentage increase in yield (approximately 72%), while soybean had the smallest (approximately 37%).

**Table 6:** Descriptive statistics of yields before and after interventions.

Statistic	Maize		Rice		Sorghum		Soybean	
	Before	After	Before	After	Before	After	Before	After
**Minimum**	0.31	0.51	1.61	2.93	0.50	0.93	0.50	1.0
**Maximum**	3.38	3.69	2.93	4.0	1.08	1.80	1.57	1.83
**Mean**	1.54	2.15	2.11	3.62	0.84	1.27	1.05	1.44
**St. Dev.**	1.06	1.24	0.72	0.60	0.30	0.47	0.41	0.31
**Skewness**	1.09	-0.01	1.61	-1.71	-1.26	1.53	-0.10	-0.34

Of the included studies, 44% reported on increases in income levels of intervention beneficiaries. Out of this number, seven studies provided actual numerical evidence of increases in income levels after a given intervention, whereas the remaining studies only indicated percentage increase in incomes without stating the base. Of those that reported good numerical information on increases in incomes, the unit of measurement was heterogeneous or inconsistent, making statistical analysis for similar crops difficult. For instance, some case studies reported increases in income levels of farmers after the intervention on a per hectare or acre basis (using prevailing market values rather than actual sales revenue), while others reported increases on a month or annual basis. Overall, six of the studies reported over 100% increases in incomes of beneficiary farmers. For example, on a per crop basis, average income levels of maize, rice and soybean farmers increased by 148% (from US$ 283 to US$ 701.9), 104% (from US$ 254 to US$ 519.25), and 119% (from US$ 264 to US$ 579.95) per ha, respectively. On average, increases in income levels of women were lower than those of men, with increases between 39% and 69% for women and 46% to 70% for men in maize- and rice-based interventions.

### Impact over time

**Fig 5:**
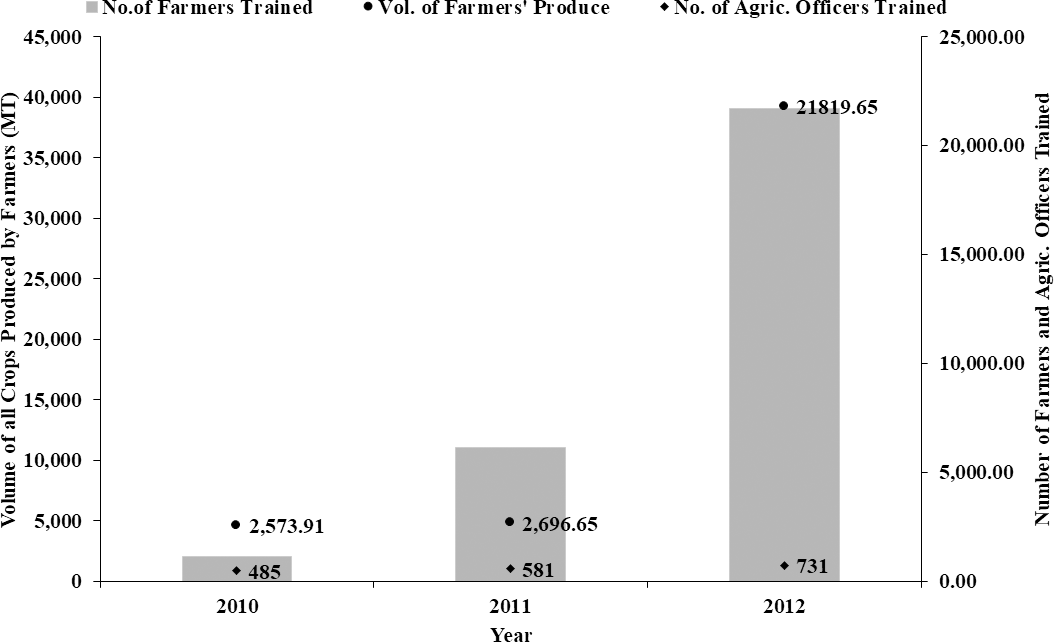
Cumulative production for all crops supported by the NRGP programme (primary axis) and cumulative number of farmers trained by the programme from 2010 to 2012.

Majority of the included studies lacked temporal and homogenous data to enable analysis of impact over time. Such data could be obtained from only 3 reports of the included studies and these were reports of the Northern Rural Growth Programme (NRGP). For 2010 to 2012, the NRGP reported over 1200%, 600% and 50% increases in production, number of farmers and agricultural officers trained, respectively (Fig 5), demonstrating a positive impact of the intervention on these indicators. There was a concomitant increase in production, and increase in number of trained farmers and officers suggesting that training, both to Agricultural Extension Agents (AEAs) and farmers might have been responsible for the improved food production under the NRGP project (Fig 5).

## Discussion

The intricate link between poverty and food insecurity has made agricultural interventions an attractive tool for international development cooperation for simultaneously reducing poverty and food insecurity [6]. Few studies have systematically evaluated the impacts of agriculture-related development interventions on food security outcomes at national or subnational scales in low income countries [5, 6]. The abundance of agriculture-related development interventions in Northern Ghana [3] raises the imperative for a systematic evaluation of their pooled effect on food security. The current study aimed at distilling the evidence for the net effect of agriculture-related interventions on household food security in Northern Ghana. Clearly, a large number of interventions (costing millions of dollars) have been implemented from 2006 to 2016 in Northern Ghana, especially the zone covering districts from latitude 8° N in Ghana. A large proportion of these interventions has been implemented or financed by development agencies and NGOs, with considerable spatial and temporal overlaps between interventions. Unfortunately, it was realized from the current study that the large volume of documents on these interventions are scattered, difficult to access and are not amenable to quantitative analysis to synthesize the pooled effect of these interventions on food security. Hence, only 22 studies were barely sufficient for the current study (albeit not for robust quantitative analysis). For the most part, the interventions were based on either prior needs assessment or pre-existing information on levels of poverty and food insecurity. The intervention designs hardly embodied quantitative impact assessment considerations and resembled direct aid to the target group. As a result, the interventions lacked the inclusion of counterfactuals or a measurement of the conditions before and after the intervention. This makes quantitative assessment of the outcomes challenging as observed by [6].

While the intervention documents claimed having impacted positively on food security, the evidence was weak and largely assumed. Because of the nature of the intervention design (as indicated earlier), it was observed that successful implementation (process goals) was often equated to impacts on food security (outcome goals), but the evidence is intuitive rather than verifiable. For example, the Innovation for Rural Prosperity project by CIDA (study code no. 50; S1 Table) based its claims of impacts on food security on the fact that farmers within the project target areas could access credits to purchase key inputs and access to trained extension personnel and quality extension services. While it is plausible that such conditions can improve household food security through increased yields or revenues, there is little evidence (if any) to indicate exactly how or what aspect of food security was improved and the scale of this improvement. This style of design and reporting, which was common to most of the included studies, seems to be the result of the expectations or internal logic of development-oriented interventions, or the inadequacy of project design.

To further illustrate this, while 82% of the included studies focused on input supply as a strategy, only 14% reported measurement of changes in yield and 41% claimed having increased levels of production. About 42% of the included studies used market access as a strategy but 44% of the studies claimed having increased revenues of intervention beneficiaries (with only seven studies providing actual numerical evidence of increases in income levels of intervention beneficiaries). In addition, even though malnutrition was inherent in the motivation for most of the interventions, none of the included studies reported on changes in nutrition of target beneficiaries. Finally, there is an issue of the resilience of the intervention to the rapidly changing and harsh ecological conditions of Northern Ghana. While this region has savannah or sahelian conditions, with predominantly rain-fed agriculture, very few interventions focused on irrigation and storage facilities. This suggests that even where interventions are effective within the duration of the project, the beneficiaries might be vulnerable to variations in climatic and market conditions, potentially resulting in erosion of gains made during the life of the intervention. It is difficult to entirely dismiss the contribution of these interventions on poverty reduction and food security. In parts of Northern Ghana, poverty dropped from 72.9% in 2006 to 44.4% in 2013, yet the three Northern regions in general continue to have the highest poverty rates in Ghana with the Northern region in particular experiencing the lowest rate of poverty reduction [12]. Hence, it is plausible that these interventions might have contributed to reductions in poverty and food insecurity but there is the need for robust assessment to clearly isolate their contributions. It is therefore important that interventions focus not only on measuring process goals but also measuring some outcome goals which would be relevant for evaluation of impacts on food security. For example, while increase in yield or revenue might be a good proxy indicator, measurement of actual availability of or accessibility to food at the household would be a stronger indicator of impact on food security [6]. This notwithstanding, increases in yields and/or incomes of farmers might positively affect food security beyond the household level. Summaries of some of the included studies, their claims or results and brief discussion or observations are presented as part of S1 Table, to illustrate the issues raised above. These case studies were selected based on the different aspects of intervention strategies and/or food security outcomes they cover, as well as the extent to which they illustrate the aforementioned issues.

For the few studies that reported changes in yield and income before and after intervention, parametric test (Pearson product moment correlation coefficient) was used to determine whether there were significant relationships between income prior to and after implementation of food security intervention, as well as the yield before and after intervention. There were strong relationships between income before and after implementation, and likewise between yield before and after implementation of food security intervention. What this suggests is that, although incomes increase for beneficiaries, low income households continue to earn relatively low incomes even after interventions, suggesting that currently food security interventions may be disproportionately benefitting those who are already outside the critical poverty level. Indeed, there are direct and strong links between interventions, prices and incomes, especially in shallow or thin markets, which are characterised by low transaction volumes, high price volatility and high tendencies for market manipulation. In shallow markets, changes in yields or level of production due to agricultural interventions can substantially alter food prices, and thereby the revenues of net food sellers and disposable incomes of net food buyers. When food prices fall due to increased production, net food buyers gain and the reverse is true for net food sellers. This should be considered carefully by interventions that aim to increase production without connections to deep markets. Generally, the few studies that provided numerical evidence of changes in incomes show that the interventions, arguably, increased the revenues of beneficiary farmers. However, it is not clear from these studies if the observed increases were as a result of higher yields per unit area, larger production volume from expansion in cultivated land area, or sale of produce at a period of higher market prices or access to ready market with guaranteed prices. These are issues worth considering to guide future interventions and policy decisions.

Another concern with the interventions that reported yield change measurements is the lack of articulation of confounding factors. Theoretically yield may be affected by both biotic and abiotic factors [13]. It is well documented that temperature and solar radiation [14, 15], rainfall frequency and amount [16], crop type [17], crop water requirement and watering regime [18] as well as water use efficiency [19] individually or jointly affect yield. It has also been suggested that seasonality [20], drought [21], crop density [22], root system traits [23] as well pests and diseases [24], and crop management [25] among others potentially influence yield. Given that the empirical materials used in the present review spanned 2006–2016, it is possible that there were spatio-temporal heterogeneities in most of these factors which could potentially affect yield. If some seasons or years, for example, experienced significantly less rainfall or poorly distributed rainfall, then an intervention, not involving irrigation, was less likely to be successful. This suggests that there was the need for the original or empirical materials to have discussed these factors in relation to crop types. However, none of the studies retrieved for this review discussed any of these confounding factors in detail. Beyond the biotic and abiotic factors, it has also been documented that economic factors such as price regimes and market mechanisms could potentially influence crop production [26, 27]. The relationships are not linear and indicates a complex interplay among biotic, abiotic, economic factors and yield. For example, regarding market mechanisms, the period for this review coincided with Ghana’s fertilizer subsidy program which started in 2008 [28], and was temporarily suspended between 2012 and resumed in late 2014. While it is uncertain how the fertiliser subsidy programme was implemented in the areas where these interventions took place, it is important that the impact of interventions are delineated from potential impacts of the fertiliser subsidy program. Rapid expansion of Ghanaian economy between 2010 and 2013 [29] could have also impacted labour mobility between agricultural and non-agricultural sectors and depending on the direction of the labour movement, this could have had adverse or positive impact on production. For example, in 2013, the Ghanaian economy was growing at approximately twice the rate as it was in 2015 [29] and this may have positively impacted production in 2013. Perhaps more importantly, these confounding factors, when considered independently or jointly, point out how complicated and complex agricultural interventions could be in terms of designing, analysis and delineating the effects of the interventions itself from other background factors.

The results in the present study also suggest that the duration of the food security intervention matters, at least for the rate of adoption of technology and increase in production levels due to extension training and capacity building. However, duration, frequency and continuity affect intervention content and fidelity of intervention delivery. While a shorter duration intervention may have lower cost, it may not be effective in the typical situation in Northern Ghana. The present study suggests that food security interventions that target extension training and increased production levels should have at least 3–5 years’ duration to be effective, contrary to the typical durations of less than three years in current development interventions (Tables 4 and 5). There is a tendency among government and development partners to seek quick solutions to the complex challenges of food insecurity. In cases where project lifespan is short, the Government should develop the capacity and interest to take over or sustain successful interventions.

Only two studies disaggregated the data based on gender of household heads. Owing to this, an analysis by gender of the dynamics of household food security could not be provided. However, of the reports that provided gender disaggregated data, women received lower revenues from intervention projects. This lower increase in incomes for women was attributed to lower yields and lower adoption of new technologies, and higher production cost compared to their male counterparts. It is known that men and women have different coping and adaptive capacities for adopting agricultural technologies due to disparate access to productive resources and socio-economic responsibilities [30].

## Conclusions

Ghana has made impressive progress in achieving food security. However, food insecurity and poverty are rife in Northern Ghana. Despite the evidence for large number of interventions implemented in Northern Ghana from 2006 to 2016, it is difficult to quantify the effect size of these interventions on food security using documents on the interventions. This by no means implies that the interventions have had no impacts; only that the pooled net effects cannot be quantitatively evaluated based on available information. The interventions ranged from input supply, market access, infrastructure development, value addition and processing, and extension training or capacity building. Most of the interventions, by nature of their designs, excluded appropriate counterfactuals and reported process goals as outcome goals, and thereby rendered quantitative evaluations unfeasible. While there was some evidence for contributions to food security, claims of impacts on food security were largely weak or assumed. Longer duration of intervention appeared to be important for effectiveness, especially for interventions that use extension training as a strategy. Moreover, given the ecological and socio-economic conditions of Northern Ghana, interventions targeting the development of water and irrigation, as well as storage facilities could be more appropriate and have more enduring benefits. Considering the low ratio of included to screened studies, general conclusions from the current study should be moderated with care.

For future policy and intervention design, measurable food security indicators should be agreed upon a priori by the Ghana Government and implementing organizations to enable consistent monitoring and evaluation of interventions. This should potentially synchronise and harmonise the food security measures used in evaluation across interventions and eventually lead to better policy outcomes. Also, given the ecological conditions and production characteristics in the regions of this study, Government and development partners should come out with an intervention framework focusing on priority areas including water and irrigation, climate change adaptation and mitigation measures, food storage facilities or processing capacity, in addition to extension and capacity building. A centralized monitoring of such interventions in Northern Ghana would be necessary to limit spatial and temporal overlaps in interventions and to minimize doubling of efforts and costs. This should also facilitate the incorporation of principles of open data or information access in food security interventions to ease objective assessment of impacts and value addition to data. To this end, a central data/information repository by Government of Ghana would be appropriate to facilitate tracking and evaluation of interventions and value addition through wider use of data.

## Supporting information

S1 Table List of projects included in the review after screening processes and summaries of selected case studies. (DOCX)

S1 File PRISMA flow chart. (DOCX)
