# Supplementary material for: Systematic review of the effects of agricultural interventions on food security in northern Ghana
Source: PLoS One. 2018 Sep 7;13(9):e0203605. doi: 10.1371/journal.pone.0203605 (PMC6128573; doi:10.1371/journal.pone.0203605)
Supplement: S1 Table — (DOCX) [file pone.0203605.s001.docx]

**Supplementary Table 1:** List of projects included in the review after screening processes

| **Study  code** | **Project or**  **Report Title** | **Year of Report** | **Duration of**  **Project** | **Organisation(s)** |
| --- | --- | --- | --- | --- |
| 1 | Expanding Climate Change Resilience in Northern Ghana (ECCRING) Project | 2014 | 2012 - 2014 | Canadian Hunger Foundation, CHF/ Association of Church Development Projects, ACDEP |
| 3 | Agricultural Development and Value Chain Enhancement Project (ADVANCE) | 2015 |  | USAID - ACDI/VOCA & ACDEP |
| 46 | Food Security and Environment Facility |  | 2008-09-16 - 2016-11-30 | CIDA |
| 47 | Food Security Through Cooperatives in Northern Ghana |  | 2013-03-28 - 2018-03-29 | CIDA |
| 50 | Innovation for Rural Prosperity |  | 2012-07-31 - 2017-10-31 | CIDA |
| 54 | Partnership for Enhancing Food and Economic Security for the Rural Poor |  | 2011-05-06 - 2016-03-31 | CIDA |
| 56 | Resilient and Sustainable Livelihoods Transformation in Northern Ghana |  | 2012-08-29 - 2015-07-31 | CIDA |
| 63 | Systems Approach to Improving and Sustaining Food Security in West Africa |  | 2012-04-24 - 2017-08-30 | CIDA |
| 67 | Transitional Support for the Food and Agriculture Sector Development Policy |  | 2002-10-01 - 2012-05-29 | CIDA |
| 106 | The project for sustainable development of rain-fed lowland rice production | 2014 | 2009-2014 | JICA/ MoFA |
| 123 | Reducing gender gaps in agriculture– a story from ADVANCE Ghana |  | 2013 -2018 | USAID |
| 135 | Annual Progress Report-2010 | 2011 | 2009 - 2016 | NRGP - MOFA/IFAD/AfDB |
| 136 | Annual Progress Report-2011 | 2012 | 2009 - 2016 | NRGP - MOFA/IFAD/AfDB |
| 137 | Annual Progress Report-2012 | 2013 | 2009 - 2016 | NRGP - MOFA/IFAD/AfDB |
| 145 | USAID RING Annual Report 2015 | 2015 | 2014 – 2019 | USAID |
| 146 | USAID RING Annual Report 2016 | 2016 | 2014 – 2019 | USAID |
| 158 | FY14 Annual Report: FTF USAID Agriculture technology transfer project | 2014 | 2011 - | USAID |
| 159 | FY2014 Annual Report (ADVANCE II) | 2014 |  | USAID |
| 170 | Do all roads lead to market: learning from AGRA's market Access programme  Case study 11: Ghana Arzakinmu "our wealth" programme | 2013 | 2010 - 2012 | Royal Netherlands Tropical  Institute (KIT) and AGRA |
| 177 | Development of market access and postharvest services (DMAPS) for smallholder farmers in the Brong-Ahafo region. | 2014 | 2011-2014 | AGRA/Concern Universal |
| 182 | Pathways to Empowerment: Increasing Food Security for 50,000 Women Farmers | 2016 |  | CARE International |
| 183 | Pathways to Resilient Livelihoods | 2016 | 2014 - 2016 | CARE International |

*Reporting year and duration of project were not evident in some of the project reports*

**Summaries of selected case studies**

1) **The *‘Food Security and Environment Facility’*** project by CIDA (*study code no. 46*) targeted increasing farmers’ productivity and environmental management. The intervention strategy was capacity building via the provision of funding for local NGOs and private sector businesses to deliver a suite of agricultural innovations and technologies to farmers. Relevant technologies included (i) improved practices for the production of food crops such as maize, soybean, groundnut, mangoes and onions; (ii) drip irrigation and dry season farming, and (iii) improved crop storage. The study reported impressive results, including: (i) substantial increase in yields of maize and soybean; (ii) high adoption of the innovations and technologies introduced (amounting to 45,045 beneficiaries, of which 25,710 were women); (iii) significant increases in farmers’ incomes due to the ability to delay sales beyond the harvest; (iv) and a reduction in the annual “hunger period”, during which daily meals are reduced due to food and income scarcity. These claims were presented in project highlights without full progress or evaluation report. Baseline conditions or variables were not reported and the margin of increase in yield was not reported. Hence, it is difficult to draw strong conclusions regarding impact on food security from this study.

2) **The *Innovation for Rural Prosperity* project by CIDA** (*study code no. 50*) delivered targeted investments in training and development of private sector enterprises providing smallholder-oriented products and services. As a result of this intervention, (i) a general marketing program regarding irrigation technologies for income opportunities, greater crop diversity and off-season production was delivered to 35,164 farmers (13,714 women and 21,450 men); (ii) 18,976 farmers (of which 10,016 were females) received agronomic extension and advisory support on nursery management, land preparation and transplanting of seedlings, effective irrigation, efficient water application mechanisms, crop disease and integrated pest management through a number of training sessions and field demonstration; and (iii) 3,038 farmers (1,871 women and 1,167 men) were able to access credit annually from some rural and micro finance institutions to buy irrigation facilities and related services, as well as inputs like seed, fertilizer and pest control. The pathways to food security from these intervention strategies are obvious, but the researchers were unable to assess the actual impact on food security given the nature of the available data. Similarly, proxy indicators of impact on food security of beneficiaries for this particular study could not be derived.

3) Among other capacity building strategies**,** the ***Partnership for Enhancing Food and Economic Security for the Rural Poor*** (*study code no. 54*) was aimed at increasing food security through raising agricultural productivity and supporting beneficiaries in the establishment and management of profitable income-generating activities. As a result of the intervention, an increased number of households have consulted extension agents for advice: 61% of households (headed by men) and 60% of households (headed by women) compared to a baseline value of 0%. Yield of main crop production increased from 310 kg per hectare to 510 kg for female-headed households. The formation of Farmer Based Organizations helped raise monthly income of beneficiaries from $16.54 to $62.83 for male-headed households and from $10.72 to $31.30 for female-headed households; and 44% of the households reported improved access to diversified financial assets from a baseline of 0%. This study reports baseline values for yields and incomes and therefore improves the confidence in the results, even though the absence of a counterfactual dilutes the strength of attribution.

4) The ***MoFA-JICA Project for Sustainable Development of Rain-fed Lowland Rice Production*** (S*tudy code no. 106*) was a well-planned and detailed rice extension strategy to boost rice production. The strategy involved the development of a technical package including land development techniques, appropriate rice cultivation techniques, and the deployment of farm management support systems and extension procedures. The project targeted raising rice yields to 3.0 ton/ha. Results of the MoFA-JICA Project for Sustainable Development of Rain-fed Lowland Rice Production project achieved average yields of 3.9 ton/ha and 3.1 ton/ha for trial plots and demonstration plots, respectively in the Northern Region. These were higher than the 2018 target yield of 3.5 ton/ha set by the Ghana National Rice Development Strategy. The project had 2,221 beneficiary farmers from 2009 to 2014 in nine districts. This was a very specific and direct experiment that achieved results that can have strong attribution of effect, but it is unclear as to whether it was a scientific experiment or a social intervention.

5) The main goal of the ***Agricultural Development and Value Chain Enhancement (ADVANCE I)* project** (*study code no. 123*) was to transform and increase the competitiveness of the maize, rice and soya value chains to *achieve a greater degree of food security* in the Northern, Upper East, Upper West, Ashanti, and Brong-Ahafo regions. The main strategy of the intervention was to improve value chain actors’ access to market and finance while strengthening local production capacities, especially of women. Results (2014/15) showed that about 51% of women who attended the good agricultural practices (GAPs) trainings used improved seeds compared to 42.9% for those who did not attend; and average yield of women who used improved seeds was about 21% higher than those who did not. The implication (and for that matter pathway to food security) is that the GAPs training resulted in increased use of improved seeds which, in turn, resulted in increased yields. Further, while men always obtained higher yields across crops, marginal increases in yields of maize and soybean were higher for women than men. This is an intervention with an obvious pathway to food security and a good (proxy) indicator for food security (yield of food crops) and sensitive to women farmers. ADVANCE II (*study code no. 159*) had similar goals and built on the achievements of ADVANCE I. The ADVANCE II worked with 37,022 smallholder farmer beneficiaries via Outgrower Businesses (OBs) providing services on mechanization, inputs and output markets. Interim results suggested increased trading of maize, rice and soybean by farmers as a result of market facilitation by the project. Again, the pathways to food security (increased production and possibly incomes) can be deduced, but it would be great to measure some food security indicators at the household level, at least for some beneficiaries.

6). The overall goal of the ***Northern Rural Growth Programme*** (NRGP, *study code nos.135/136*) by MoFA/IFAD/AfDB was to contribute to an equitable and sustainable poverty reduction and food security through sustainable increase in incomes for rural households in Northern Ghana. The NRGP covers the three (3) northern regions (Upper East, Upper West and Northern) and five districts namely Kintampo North, Kintampo South, Pru Sene and Pru Districts in the Brong Ahafo Region. Progress reports of 2010 and 2011 (*study code nos. 135/136*) indicated high interest in the programme, with some of the programme components oversubscribed. Large areas were cultivated with maize and soybean. The programme reached a wide area and a large number of beneficiaries. For example, as of the end of 2010, the programme had enlisted 31,235 individual farmers (23,828 males and 7,426 females) to participate in the major season production. A total of 41,449 ha of various crops (83 % of maize, 13 % of soya and 4% of sorghum) were cultivated under the Commodity Chain Development component, while 2,028 farmers were able to access credit for the major season production. Similar impressive reach was reported in 2011. However, the outcome measures in the annual reports (2010 and 2011) do not show direct impacts on household food security even though the pathway is obvious. Hence, increase in area of production and access to credit was assumed to have a direct, positive impact on household food security.

The 2012 report (*study code* no. 137) indicated that the average yields of beneficiary farmers had increased by 150%, 160% and 58% for maize, soya and sorghum respectively. In absolute terms, maize yields had increased from 0.8 MT/ha to 2.2 MT/ha, soya increased from 0.5 MT/ha to 1.5 MT/ha whilst sorghum increased from 0.5MT/ha to 1.8MT/ha. The report indicated that farmers’ incomes almost doubled due to increase in yields, reduction in post-harvest losses, and improved access to market. As of 2015, the report (*study code no. 137*) indicated that the total area of land cultivated was lower than that of 2010 but yield increases were higher in 2015. For example, average yields were approximately 3.5 MT/ha and 2.2 MT/ha for maize and soybean, respectively. Average yield of sorghum had reached 1.9 MT/ha. Due to the integrated approach adopted by the NRGP, it is plausible that the concurrent improvement in yields and production volumes, reduction in post-harvest losses, and access to market could improve food security in the target regions and districts. However, direct measurement of household food security impact would have enabled a more robust evaluation of the programme outcomes. Beneficiaries also dropped in and out over the years, making it difficult to attribute impact on beneficiaries over time.
