# Supplementary material for: Systematic review of the effects of agricultural interventions on food security in northern Ghana
Source: PLoS One. 2018 Sep 7;13(9):e0203605. doi: 10.1371/journal.pone.0203605 (PMC6128573; doi:10.1371/journal.pone.0203605)
Supplement: S1 File — (DOCX) [file pone.0203605.s002.docx]

Records identified through online searching
(n =194)

Screening

Included

Eligibility

Identification

Additional records identified through organizational visits
(n =221)

Records after duplicates removed
(n =291)

Records screened
(n =291)

Records excluded
(n =65)

Studies assessed for eligibility
(n =226)

Full-text articles excluded, with reasons
(n =204)

- Cross country policy study;
- Coarse data;
- Review, not intervention;
- Outside scope of study;
- Policy support to Government;
- Project list;
- Project synopsis;
- Outside zone of influence;
- Multi country study
- National and regional highlights of Agriculture;
- Population survey;
- Baseline survey;
- Lacks measurable outcomes;
- Unrelated research article.

Studies included in qualitative synthesis
(n = 22)

Studies included in quantitative synthesis (meta-analysis)
(n = 0)

**S1 File**. PRISMA Flow chart.
